# Prediction of age and brachial-ankle pulse-wave velocity using ultra-wide-field pseudo-color images by deep learning

**DOI:** 10.1038/s41598-020-76513-4

**Published:** 2020-11-09

**Authors:** Daisuke Nagasato, Hitoshi Tabuchi, Hiroki Masumoto, Takanori Kusuyama, Yu Kawai, Naofumi Ishitobi, Hiroki Furukawa, Shouto Adachi, Fumiko Murao, Yoshinori Mitamura

**Affiliations:** 1 Department of Ophthalmology, Tsukazaki Hospital, Himeji, Japan; 2 Department of Technology and Design Thinking for Medicine, Hiroshima University Graduate School, Hiroshima, Japan; 3 Department of Ophthalmology, Institute of Biomedical Sciences, Tokushima University Graduate School, Tokushima, Japan; 4 Department of Cardiology, Tsukazaki Hospital, Himeji, Japan

**Keywords:** Risk factors, Predictive markers

## Abstract

This study examined whether age and brachial-ankle pulse-wave velocity (baPWV) can be predicted with ultra-wide-field pseudo-color (UWPC) images using deep learning (DL). We examined 170 UWPC images of both eyes of 85 participants (40 men and 45 women, mean age: 57.5 ± 20.9 years). Three types of images were included (total, central, and peripheral) and analyzed by k-fold cross-validation (k = 5) using Visual Geometry Group-16. After bias was eliminated using the generalized linear mixed model, the standard regression coefficients (SRCs) between actual age and baPWV and predicted age and baPWV from the UWPC images by the neural network were calculated, and the prediction accuracies of the DL model for age and baPWV were examined. The SRC between actual age and predicted age by the neural network was 0.833 for all images, 0.818 for central images, and 0.649 for peripheral images (all *P* < 0.001) and between the actual baPWV and the predicted baPWV was 0.390 for total images, 0.419 for central images, and 0.312 for peripheral images (all *P* < 0.001). These results show the potential prediction capability of DL for age and vascular aging and could be useful for disease prevention and early treatment.

## Introduction

Vascular aging is one of the most important characteristic changes of the aging process^1,2^. It is assessed based on the structural and functional arterial properties and is generally considered to be an independent predictor of cardiovascular risk^3^. Pulse-wave velocity (PWV), which is the velocity at which the pulse wave of the aorta generated by the pulsation of the heart propagates toward the periphery, is an index of systemic arterial stiffness and vascular disorders. It is measured at two arterial locations in the body and calculated using the difference in distance and time between the two locations^4^. In clinical practice, PWV is typically measured at the brachial and ankle arteries, and the calculated branchial-ankle PWV (baPWV) value indicates the degree of systemic arteriosclerosis and vascular disorders^5,6^. In fact, baPWV has been reported to be associated with risk factors for many systemic diseases, including cardiovascular disease^7–10^, hypertension^11–13^, diabetes mellitus^13^, and hyperuricemia^14^.

Recently, artificial intelligence (AI) technology including deep learning (DL) has resulted in remarkable progress in medicine, and various applications for diagnostic imaging have been reported^15^. In the field of ophthalmology, many researchers, including the authors, have reported the performance of DL in image analysis using in vivo laser confocal microscopy, optical coherence tomography (OCT), OCT angiography, and ultra-wide-field fundus ophthalmoscopy^16–27^. Recently, Poplin et al. reported that using machine-learning algorithms, cardiovascular risk factors including age can be predicted from retinal fundus photographs^28^. Because the retinal blood vessels are the only blood vessels that can be directly and noninvasively observed in the human body, findings such as retinal blood vessels have been widely used in many classification systems. Some of the most globally accepted of these systems include the classification of diabetic retinopathy based on evidence from a large-scale clinical trial by the Early Treatment Diabetic Retinopathy Study^29^ and the classification of hypertensive retinopathy, including the Keith-Wagener-Barker classification and the Scheie classification^30^. Such retinal findings may show minute changes in various systemic diseases from an early stage. In their study, Poplin et al. used only fundus images with a 45° field of view to predict cardiovascular risk factors; however, such images reflect only a small portion of the entire retina. In contrast, ultra-wide-field pseudo-color (UWPC) images taken by ultra-wide-field fundus ophthalmoscopy with a 200° field of view are likely to contain more information on systemic factors than normal 45° fundus images. The use of AI to predict age and baPWV with sufficient accuracy will be extremely useful in clinical practice in terms of preventive medicine.

In this study, we investigate the potential capability of AI to predict age and vascular aging from UWPC images by examining the correlation between actual age and baPWV and predicted age and baPWV from UWPC images by DL.

## Results

A total of 170 images of both eyes of 85 participants (40 men and 45 women, mean age: 57.5 ± 20.9 years) were included in the study. The mean actual baPWV of the participants was 1.14 ± 0.09 × 10^3^ cm/s. The characteristics of the participants are summarized in Table 1. The correlation coefficient between the actual baPWV and the actual age was 0.441 (95% confidence interval [CI]: 0.245–0.637; *P* < 0.001).

**Table 1:** Participant characteristics.

	Participants (n = 85)
Age (years)	57.5 ± 20.9
Gender (male/female)	40/45
Brachial-ankle pulse-wave velocity (× 10^3^ cm/s)	1.14 ± 0.09
Systemic hypertension (%)	14 (16.5%)
Diabetes mellitus (%)	6 (7.1%)
Hyperlipidemia (%)	11 (12.9%)

**Figure 1:**
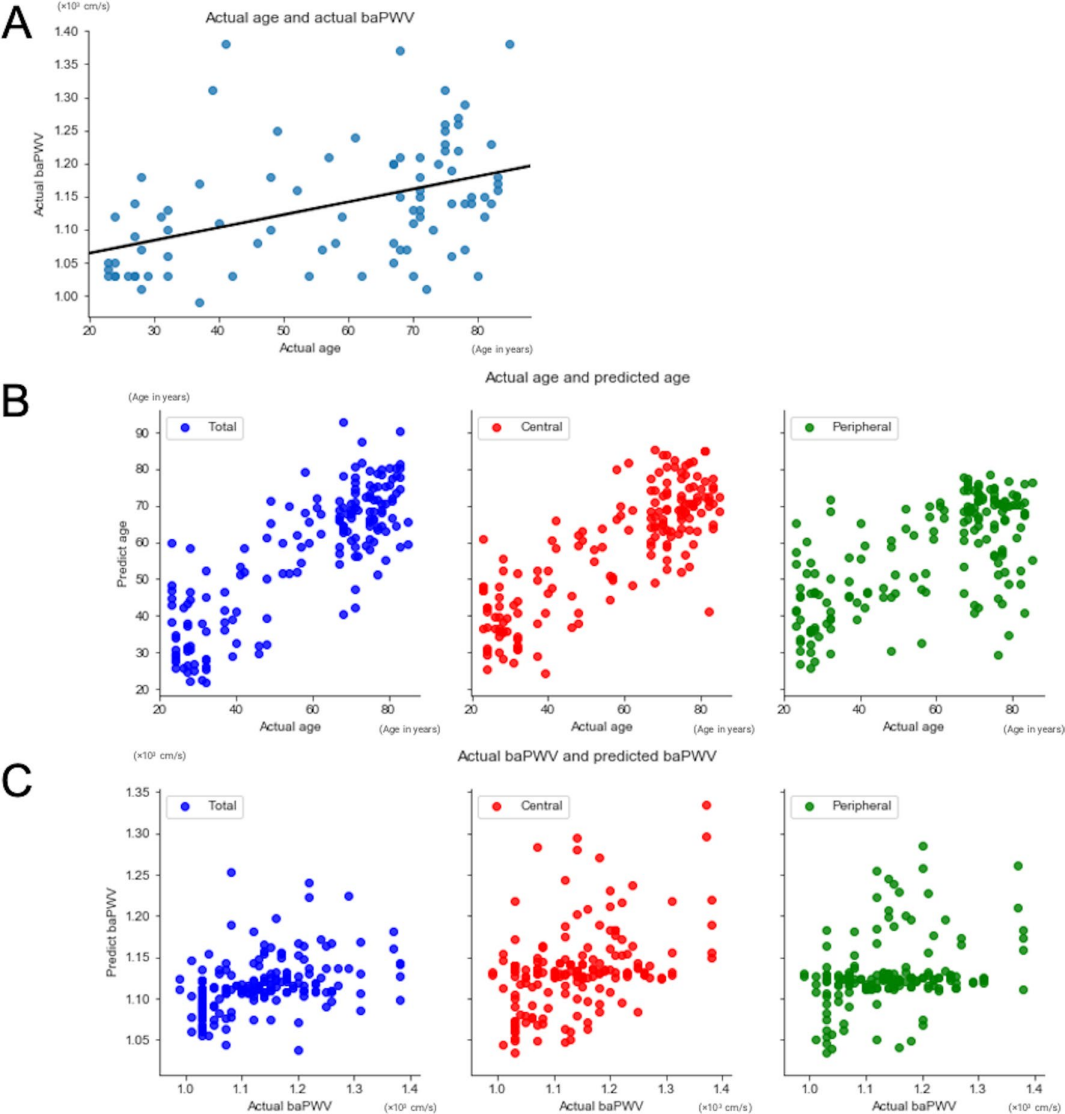
Correlation between the actual and predicted values by the neural network of age and brachial-ankle pulse-wave velocity (baPWV). **(A)** Correlation between the actual age and actual baPWV. The solid line represents the best-fit linear regression line (*y* = 0.00193x + 1.026). **(B)** Correlation between the actual and predicted ages from the ultra-wide-field pseudo-color (UWPC) images by the neural network. The figure on the left shows the correlation obtained when the total images were used for the prediction. The figure in the middle shows the correlation obtained when the central images were used. The figure on the right shows the correlation obtained when the peripheral images were used. **(C)** Correlation between the actual and predicted baPWV from the UWPC images by the neural network. The figure on the left shows the correlation obtained when the total images were used for the prediction. The figure in the middle shows the correlation obtained when the central images were used. The figure on the right shows the correlation obtained when the peripheral images were used.

Table 2 shows the predicted baPWV and age using each type of UWPC image (total, central, and peripheral) by the neural network. When the neural network made its prediction from the UWPC total images, the standard regression coefficient (SRC) between the predicted age and the actual age was 0.833 (95% CI 0.730–0.933), and the SRC between the predicted baPWV and the actual baPWV was 0.390 (95% CI 0.217–0.559). When the prediction was made from the UWPC central images, the SRC between the predicted age and the actual age was 0.818 (95% CI 0.718–0.921), and the SRC between the predicted baPWV and the actual baPWV was 0.419 (95% CI 0.249–0.593). When the prediction was made from the UWPC peripheral images, the SRC between the predicted age and the actual age was 0.649 (95% CI 0.500–0.803), and the SRC between the predicted baPWV and the actual baPWV was 0.312 (95% CI 0.140–0.490; all *P* < 0.001; Fig. 1; Table 2).

**Table 2:** Correlation between actual and predicted values of age and brachial-ankle pulse-wave velocity.

	Predicted age (years)	SRC^a^ (95% CI)	*P* value^c^	Predicted baPWV (× 10^3^ cm/s)	SRC^b^ (95% CI)	*P* value^d^
Total image of UWPC	57.0 ± 17.5	0.833 (0.730–0.933)	< 0.001	1.12 ± 0.03	0.390 (0.217–0.559)	< 0.001
Central image of UWPC	57.1 ± 14.3	0.818 (0.718–0.921)	< 0.001	1.13 ± 0.04	0.419 (0.249–0.593)	< 0.001
Peripheral image of UWPC	58.7 ± 15.8	0.649 (0.500–0.803)	< 0.001	1.13 ± 0.05	0.312 (0.140–0.490)	< 0.001

*baPWV* brachial-ankle pulse-wave velocity, *CI* confidence interval, *SRC* standardized regression coefficient, *UWPC* ultra-wide-field pseudo-color.

^a^SRC between predicted and actual age.

^b^SRC between predicted and actual baPWV.

^c^Significance of correlation between predicted and actual age.

^d^Significance of correlation between predicted and actual baPWV.

**Figure 2:**
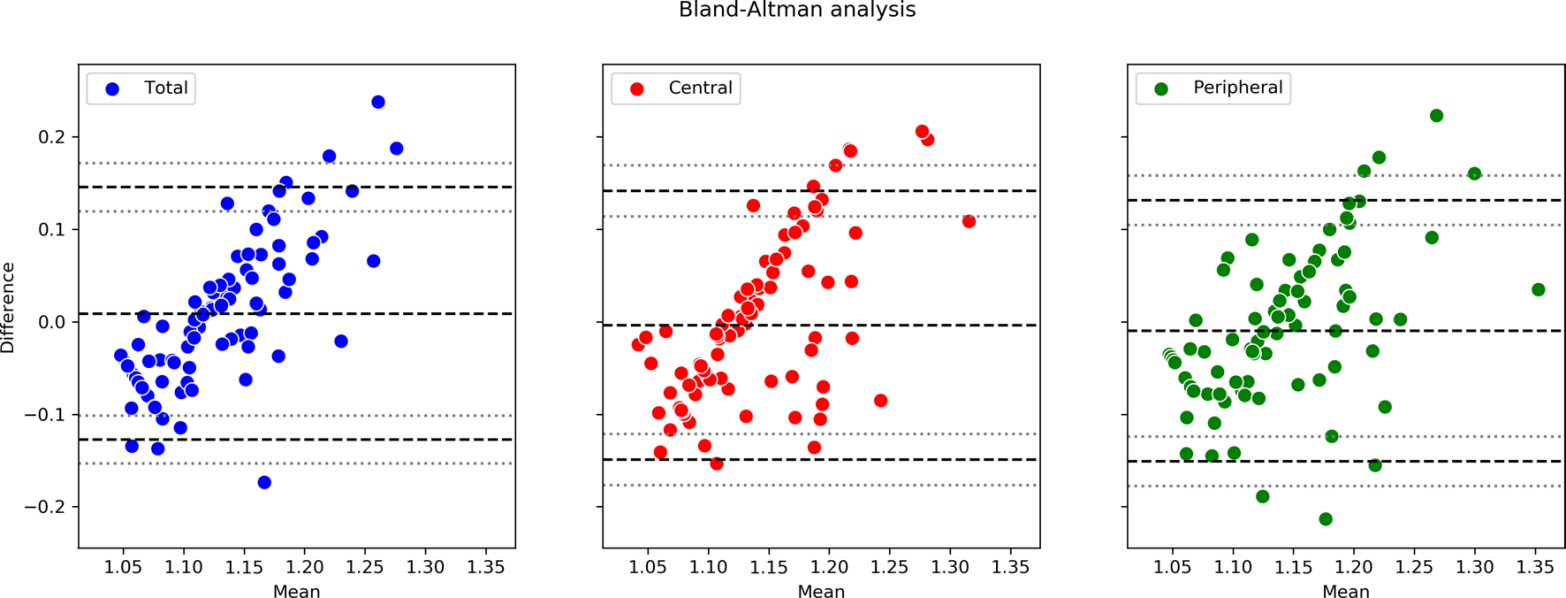
Bland–Altman plots between predicted brachial-ankle pulse-wave velocity (baPWV) and actual baPWV. The horizontal axis is "the average values of the predicted and actual baPWV," and the vertical axis is "the difference between the predicted and actual baPWV". The three black dashed lines in each plot indicate the upper LOA, the mean, and the lower LOA from top to bottom, and the gray dotted lines above and below each LOA indicate its 95% confidence interval. The figure on the left shows the total images, the figure in the middle shows the central images, and the figure on the right shows the peripheral images used for the prediction. There was no significant difference in the additional error, but proportional error was significantly observed in all the plots.

Bland–Altman plots showed good agreement between "the average values of the predicted baPWV and actual baPWV" and "the difference between the predicted baPWV and actual baPWV" with narrow limits of agreement (LOA) (Fig. 2). Regarding the UWPC total images, the average was 0.010, the upper 95% LOA was 0.146 (95% CI 0.120–0.171), the lower 95% LOA was -0.127 (95% CI: -0.153– -0.102), and the correlation coefficient was 0.775 (95% CI 0.692–0.838) (P < 0.01). In case of the UWPC central images, the average was − 0.004, the upper 95% LOA was 0.142 (95% CI 0.114–0.169), the lower 95% LOA was − 0.149 (95% CI − 0.176 to − 0.121), and the correlation coefficient was 0.637 (95% CI 0.516–0.732) (P < 0.01). With regard to the UWPC peripheral images, the average was − 0.010, the upper 95% LOA was 0.132 (95% CI 0.105–0.158), the lower 95% LOA was − 0.151 (95% CI − 0.178 to − 0.124), and the correlation coefficient was 0.534 (95% CI 0.392–0.651) (P < 0.01). In all the plots, there was significant difference in the proportional error because each correlation coefficient was significant.

**Figure 3:**
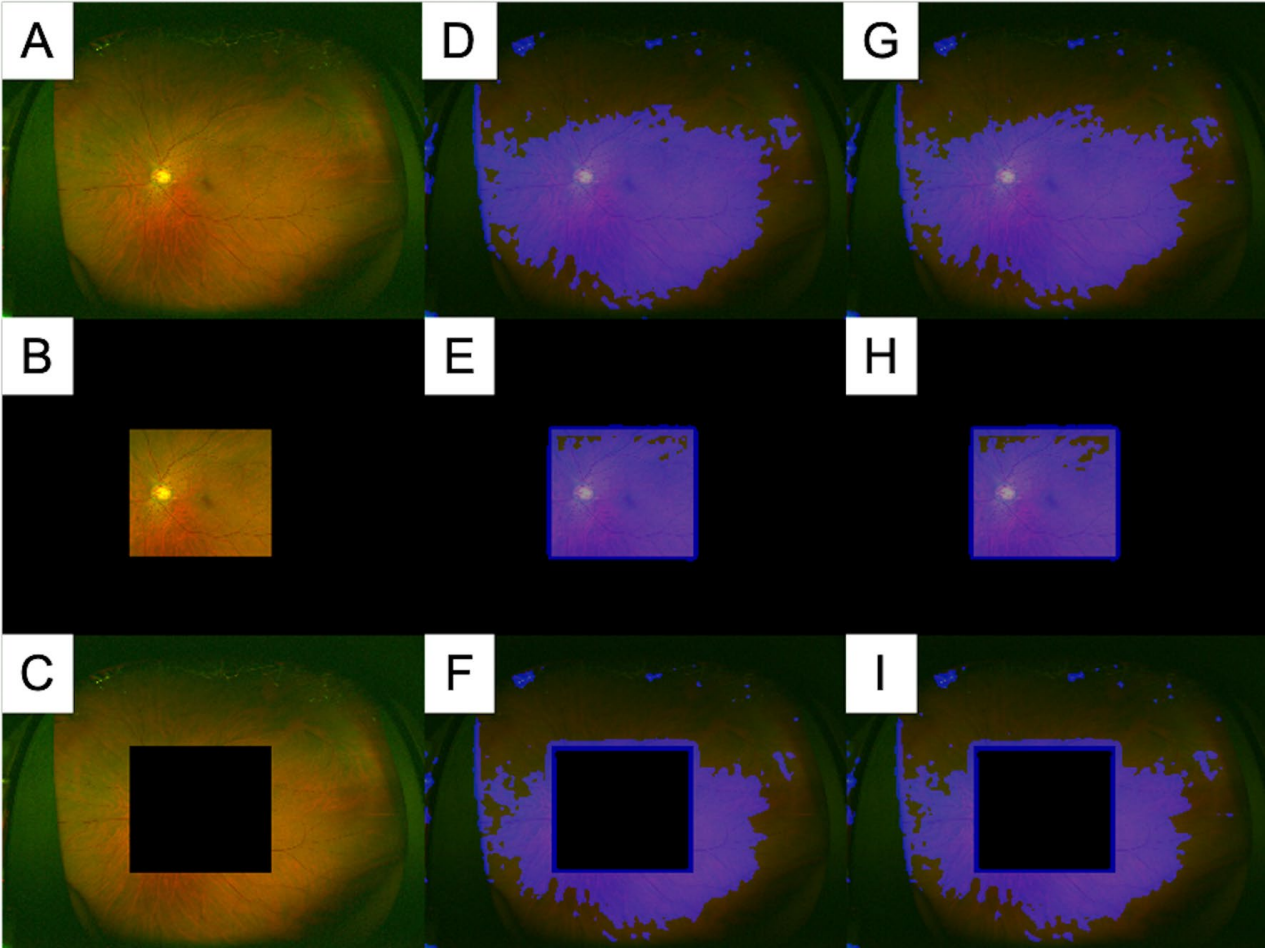
The composite images produced by the heat maps when predicting branchial-ankle pulse-wave velocity and age were superimposed on three representative types of UWPC images: total, central, and peripheral images. **(A)** Total UWPC image, **(B)** central UWPC image, **(C)** peripheral UWPC image **(D–F)** The composite images produced by the heat maps when predicting branchial-ankle pulse-wave velocity was superimposed on each type of UWPC image. **(G–I)** The composite images produced by the heat maps when predicting age were superimposed on each type of UWPC image. In **(D,E,G,H)**, it is evident that the neural network model focuses on the posterior pole area, including the optic nerve head. On the other hand, in **(D,F,G,I)**, the neural network model tends to focus on parts other than the periphery.

Figure 3 displays the composite images produced by heat maps when the predictions of baPWV and age were superimposed on three types of UWPC images (total, central, and peripheral). Blue indicates the intensity of the neural network-based identification. In predicting both age and baPWV, the neural network mainly placed its focus on the posterior pole, including the optic nerve head and vascular arcade, in both the total images and central images. Moreover, the network did not focus on peripheral images or the peripheral part of the total images.

## Discussion

The results of this study demonstrate that using UWPC images, DL can predict age with high accuracy. In addition, the predicted baPWV from UWPC images using DL was significantly correlated with the actual baPWV, although the correlation was small, indicating the DL’s potential capability to predict vascular aging. In this study, the included images were normal fundus images without apparent retinal findings such as retinal hemorrhage, hard exudate, and soft exudate. This means that, interestingly, DL was likely capable of predicting age with a high degree of accuracy from fundus images that had no apparent abnormal clinical findings and also recognized minute differences in retinal structures, including retinal blood vessels, thereby estimating the severity of arteriosclerosis and vascular disorders.

Arteriosclerosis that causes cardiovascular disease can progress to arterial occlusive disease, which may not only be fatal but also have serious sequelae. However, addressing risk factors early and promoting healthy lifestyle changes can improve this condition^31,32^. Moreover, ophthalmologists can reliably detect the risk of various systemic diseases, including diabetes mellitus and hypertension, from fundus photographs, when retinal hemorrhage, hard exudate, or soft exudate is found in the photographs of patients with moderate or higher hypertensive retinopathy^30^ or in the photographs of patients with initial stage or higher diabetic retinopathy^29^. However, identifying such risks using fundus images is challenging for ophthalmologists when patients have mild or less hypertensive retinopathy with minute changes in the retina or when patients with diabetes mellitus have not developed clear diabetic retinopathy. However, as shown in our results, the fact that DL was able to predict baPWV, the value that reflects the status of blood vessels in the entire body, relatively easily from apparently normal UWPC images suggests that it can be used as an assessment item in screening tests.

Although they did not present the specific results, Poplin et al.^28^ claimed that there was a fairly linear relationship between actual age and the DL’s predicted age from fundus images with a 45° field of view. In our study, the SRCs between the actual age and the DL’s predicted age from the UWPC images with a 200° field of view were 0.833, 0.818, and 0.649 for total, central, and peripheral images, respectively. Our results cannot be simply compared with the results from the study by Poplin et al.; however, the results of both studies demonstrate the DL’s ability to predict age with very high accuracy from UWPC images with 45° and 200° fields of view. For the baPWV values, the SRCs between the actual and the DL’s predicted values were 0.390, 0.419, and 0.312 for total, central, and peripheral images, respectively, which indicate that the prediction accuracy for age is clearly higher than that of baPWV. This means that DL predicts age more accurately than baPWV by detecting other age-related changes in the fundus in addition to the vascular changes from UWPC images.

One of the age-related changes observed in the fundus is a decrease in choroidal thickness. In normal adult eyes, the choroidal thickness has been reported to decrease by 1.1 to 4.1 µm per year with aging^33,34^. When the choroid becomes thinner, the choroidal vessels become more visible, and the tigroid fundus can be clearly observed^35,36^. In fact, it has been reported that the degree of choroidal thinning can be predicted based on changes in the tigroid fundus^37^. In the heat maps produced when the neural network predicted age, the model mainly placed its focus on the posterior pole, including the optic nerve head and the retinal vascular arcade, in the UWPC total and central images. With aging, the neuroretinal rim area decreases by 0.28% to 0.39% per year, and the mean ratio of the cup/disk diameter increases by about 0.1 between the age of 30 and 70 years^38^. Another age-related change is a decreased number of retinal ganglion cells histologically. As a result, the retinal nerve fiber layer becomes thin, and the number of retinal pigment cells decreases. Moreover, the pigment of the retinal pigment epithelium becomes thin, with an associated decrease in retinal reflexes^39^. When a neural network predicts age from UWPC images, the network is likely to detect not only changes in the blood vessels but also the aforementioned age-related changes, including morphological changes in the optic nerve head, changes in retinal color tone, and changes in the permeability of choroidal blood vessels, thus predicting age more accurately than baPWV.

Another point to note in our results was that for the prediction of age, the strongest correlation was observed when the prediction was made from the total images, whereas for the prediction of baPWV, the strongest correlation was obtained from the central images. In general, having more information results in a more accurate AI prediction. Therefore, the authors expected that the predictions using the total images would produce the highest prediction accuracy for both age and baPWV predictions. With regard to retinal blood vessels in older individuals with arteriosclerosis, it is known that the diameters of the blood column of the retinal arteries are significantly irregular as compared with normal subjects^40^. However, retinal blood vessels in the peripheral retina are thinner than in the posterior retina. To perform the analysis in our study, all UWPC images were downsized from 3900 × 3072 pixels to 256 × 192 pixels. This image downsizing may have prevented the neural network from fully identifying minute changes in the peripheral retinal blood vessels. These possible reasons may explain why, for the prediction of age, the strongest correlation was observed with the total images, which had the largest amount of information, whereas for the prediction of baPWV, in contrast to our expectation, the strongest correlation was with the central images, in which the blood vessels of the posterior pole were easier to for the network to focus on. UWPC images are considered to contain more information than normal fundus images; however, the images of the posterior pole may be sufficient when predicting baPWV with the same image quality used in this study.

In a study conducted in patients with low to moderate cardiovascular risk factors, Hung et al.^41^ reported that aging was predictive of increased baPWV and baPWV was a composite risk factor for early atherosclerotic changes and a predictor for the development of diastolic dysfunction and long-term cardiovascular risk. Our study results are consistent with the report by Hung et al., indicating some correlations between age and actual baPWV. Tomiyama et al. reported that, of 12,517 patients (male, 8227; female, 4290) who received no medical treatment and had no history of cardiovascular disease, the correlations between age and actual baPWV were 0.50 in males and 0.68 in females^42^. Our study participants included those who were receiving treatment for conditions including hypertension, diabetes mellitus, or hyperlipidemia, although no obvious retinopathy was observed. The hemodynamics of the participants might have been more variable as compared with participants who received no medical treatment, which may explain why the correlations found in our results were not as high as those reported by Tomiyama et al.

One of the major limitations of our study is the small sample size. Next, to shorten the analysis time, the original UWPC image with 3900 × 3072 pixels was resized to 256 × 192 pixels. This research was conducted in a single facility. In future studies, analysis using the UWPC images from multiple facilities would be required. In addition, we excluded images for which retinal specialists would be unable to determine fundus findings because of conditions such as severe cataracts, corneal opacity, or vitreous opacity due to vitreous hemorrhage. We also excluded images with obvious retinochoroidal diseases such as diabetic retinopathy, retinal vein occlusion, and age-related macular degeneration. Furthermore, it is known that age and PWV do not always have a significant correlation in the population of adults older than 70 years, as there are many complications of overt and latent chronic diseases. Moreover, there are large differences in the progress of arteriosclerosis among individuals^43^. Future studies are needed to examine how AI can predict age and baPWV from UWPC images in patients with various retinochoroidal diseases as well as in the elderly population. However, the ability to predict baPWV from fundus images before the onset of fundus diseases would be highly useful from the viewpoint of preventive medicine, as baPWV is associated with risk factors of various common systemic vascular diseases.

In summary, our results indicate that DL can predict age and baPWV from UWPC images. Using AI to screen fundus images may provide useful information for assessing the early risks of various systemic diseases in asymptomatic patients. This finding is very significant from the viewpoint of disease prevention and early treatment for patients.

## Methods

### Data set

This study adhered to the tenets of the Declaration of Helsinki and was approved by the ethics committee of Tsukazaki Hospital (Himeji, Japan). Written informed consent was obtained from all participants, who were provided sufficient information regarding the nature of the study and possible outcomes.

On the same day that the participants had their UWPC image taken using the ultra-wide-field scanning laser ophthalmoscope (Optos 200Tx; Optos PLC, Dunfermline, UK), their age was registered and baPWV was measured. Individuals with apparent ocular diseases were not enrolled as study participants. Retinal specialists examined the collected fundus images, and those images with obvious fundus lesions or vitreous opacity were excluded. Data were collected between May 22, 2018, and February 14, 2019, and a total of 170 images of both eyes of 85 participants were included in the study.

From the 170 UWPC images, three types of images (total, central, and peripheral images) were created by experienced certified orthoptists. Images with no filling were referred to as UWPC total images. The UWPC central images were created by filling the peripheral region of a total image with black color to obtain the same 45° field of view, including retinal vascular arcades and optic disk reported in the study by Poplin et al.^28^, in which age was predicted from the posterior pole images taken by fundus cameras. The UWPC peripheral images were created by filling the central region of a total image with black color.

**Figure 4:**
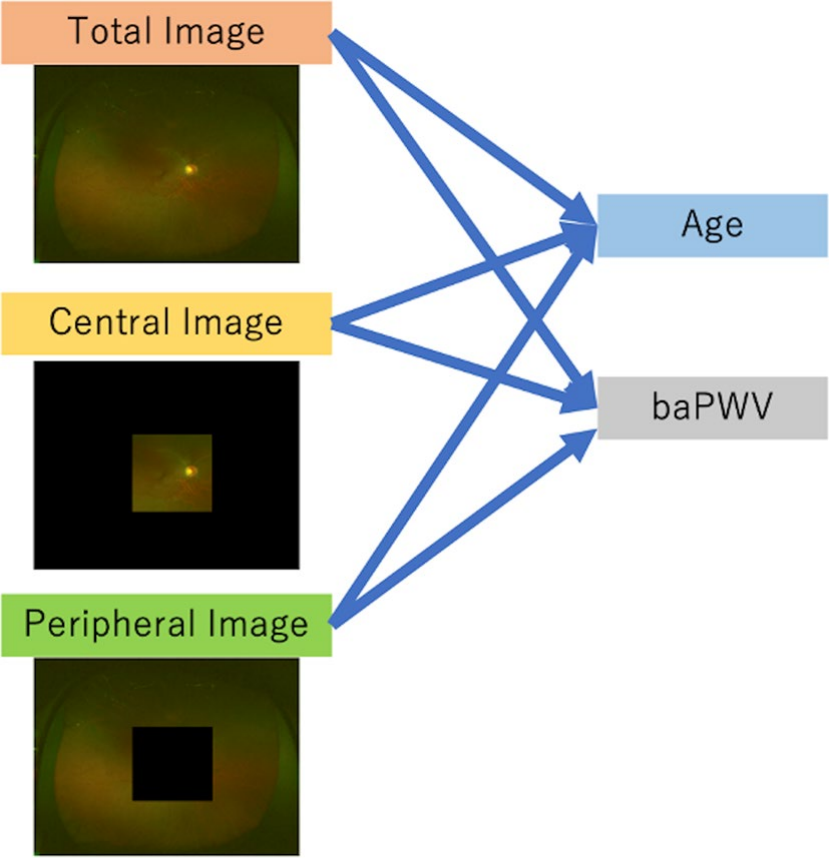
Analysis and validation of three types of ultra-wide-field pseudo-color images.

In this study, six types of validations were performed to predict baPWV and age from each of the three types of UWPC images (total, central, and peripheral images; Fig. 4).

k-fold cross-validation (k = 5) was used for these validations^44,45^. A total of 170 images of 85 people were divided into k groups in a method in which the images taken from the same participant would belong to the same group. The (K − 1) groups were used for training, and one group was used for validation.

For the training data, nine types of image processing were performed: unprocessed, two types of brightness correction, two types of gamma correction, gaussian blur, histogram equalization, and two types of noise addition. Three of the nine types of image-processing techniques were randomly employed, and the images obtained were flipped horizontally. Using such processing, the number of images used for training increased by six times. Using the amplified training data, a convolutional neural network was developed, and the validation data were used to evaluate the performance of the constructed neural network. This process was repeated k times so that all images in the k groups could be used for validation.

This method is similar to that used in the previous studies^18,21^.

### Brachial-ankle PWV measurement

The baPWV measurements were performed using the methodology described in previous studies^5,46^. The baPWV measurements were obtained using a noninvasive automated device (model BP-203RPE-III; Nihon Colin, Tokyo, Japan). After each participant had rested in the supine position for five minutes, cuffs were placed around the upper arms (brachial arteries) and the ankles (posterior tibial arteries) bilaterally to perform measurements. We used the blood pressure measured at the upper limb, and the distance between the sampling points of baPWV was calculated automatically based on the height of the participant. The path lengths from the suprasternal notch to the ankle (La) were obtained using the formula: La = 0.8129 × height (cm) + 12.328. The path lengths from the suprasternal notch to the brachium (Lb) were obtained by the formula: Lb = 0.2195 × height − 2.0734^5^. Tba was defined as the time interval between the brachium and ankle, which was measured from the wave front of the brachial waveform and that of the ankle waveform^5^. The baPWV was then calculated according to the formula: baPWV = (La − Lb)/Tba.

The baPWV values on the right side (right brachial artery and right posterior tibial artery) and the left side (left brachial artery and left posterior tibial artery) were recorded simultaneously, and the higher value of the two was used as the actual baPWV of the participant.

### DL model and its training

We used a neural network called Visual Geometry Group-16 (VGG-16)^47^. It has been reported that convolutional neural networks such as VGG-16 automatically learn local features^48,49^. All images were downsized from 3900 × 3072 pixels to 256 × 192 pixels for analysis.

VGG-16 consists of five convolutional blocks and a fully connected layer block. A convolutional block consists of convolutional layers^48–50^ that automatically detect the features in the input (image) that influence the output (in this study, age or baPWV); ReLU^51^, which is an activation function to avoid vanishing gradient problems; and a max-pooling layer^52^, which compresses the amount of information.

After five convolutional blocks, the tensor passes through a fully connected layer block consisting of a flatten layer and two fully connected layers. The flatten layer removes spatial information from the extracted features. The layer then passes through the fully connected layers to compress many extracted features. We output the final, fully connected layer without letting it pass through the activation function. We used this output as the age or baPWV to train the neural network and constructed a model with the ability to predict age and baPWV.

To accelerate the training process and improve the performance of a model, even with a small amount of data, we used a technique called transfer learning. Weights learned from an image dataset called ImageNet containing over 14 million images of 20,000 different types are often used in transfer learning. The parameters trained by ImageNet were used as the initial weights for the first four convolution blocks^53^. We used the Adam method to update the parameters for our model (learning ratio = 0.0001)^54^.

Keras (https://keras.io/ja/) of Python (backend TensorFlow) was used for the construction and validation of the neural network. The code used for the validation is provided in Dataset S1.

### Outcome

We obtained the following SRCs between the predicted values by the neural network and actual values of both age and baPWV from each type of UWPC images: SRC between predicted age from the UWPC total images and actual age (ρ11); SRC between predicted baPWV from the UWPC total images and actual baPWV (ρ12); SRC between predicted age from the UWPC central images and actual age (ρ21); SRC between predicted baPWV from the UWPC central images and actual baPWV (ρ22); SRC between predicted age from the UWPC peripheral images and actual age (ρ31); SRC between predicted baPWV from the UWPC peripheral images and actual baPWV (ρ32). We used the obtained SRCs as performance evaluation indexes.

In addition, for each type of UWPC image, Bland–Altman analysis was performed on the predicted and actual baPWV.

### Statistical analysis

Linear regression analysis was used to examine the association between actual baPWV and actual age. The SRCs between the values predicted by the neural network and the actual values of both age and baPWV from each type of UWPC image were calculated using the following method. A generalized linear mixed model (random intercept model) was constructed as follows.

$$ \begin{gathered}   {\text{pred}}\prime {\text{ }}[{\text{n}}]\sim {\text{Normal}}(\alpha [{\text{PID}}[{\text{n}}]) + \beta *{\text{ actual}}\prime [{\text{n}}],{\text{ }}\sigma _{P} ^{2} \;n = 1,2,3, \ldots ,{\text{N}} \hfill \\   \alpha \left[ {\text{k}} \right]{\mkern 1mu}  = {\mkern 1mu} \alpha _{{{\text{all}}}}  + {\mkern 1mu} \alpha _{{{\text{id}}}} {\text{ }}\left[ {\text{k}} \right]{\text{ k }} = 1,2,3, \ldots ,{\text{K}} \hfill \\   \alpha _{{{\text{id}}}} [{\text{k}}]\sim {\text{Normal}}(0,\sigma _{\alpha } ^{2} ){\text{ k}} = 1,2,3, \ldots ,{\text{K}} \hfill \\  \end{gathered}  $$

where [pred'] is the predicted value that is standardized so that it has a mean 0 and variance 1, and [actual'] is the actual value processed in the same way. [PID] is a variable that stores which data belongs to which patient.

The prior distributions of α_all and β0 were uniform distributions of (− ∞, ∞), and σ_P^2, and σ_α^2 were irregular uniform distributions of (0, ∞). The number of chains was 4, the number of times of random number generation was 2000, and the burn-in period was 1000. No decimation was performed. To generate the random number, we used the No-U-Turn Sampler, one of the implementation methods of the Hamiltonian Monte Carlo method^55^. The representative value was the posterior median value, 95% CI (2.5–97.5%). The analysis was performed using pyStan, a Python package (https://pystan.readthedocs.io/en/latest/getting_started.html). *P* values less than 0.05 (*P* < 0.05) were considered significant.

### Heat maps

Interpretable machine-learning techniques can be grouped into two categories, namely intrinsic interpretability and post-hoc interpretability^56^. Class activation method (CAM) is a post-hoc local explanation method which explains why the model makes a particular decision.

Heat maps were produced using the Score-CAM method to indicate where the neural network placed its focus^57^. Score-CAM removes the dependence on the gradients by obtaining the weight of each activation map through its forward passing score on the target class.

The target layer was the max-pooling layer of the first block. The ReLU function was used to correct the loss function during backpropagation.

## Data Availability

Fundus images and image data sets used in the study are available from the corresponding authors upon reasonable request.
